# Trajectory Patterns of Perioperative Anxiety and Depressive Symptoms in Patients With Digestive System Malignancies and Their Impact on Postoperative Outcomes

**DOI:** 10.62641/aep.v54i4.2266

**Published:** 2026-08-15

**Authors:** Yige Zhao, Mengmeng Li

**Affiliations:** ^1^Department of Gastroenterology, Zhejiang Xinan International Hospital, 314031 Jiaxing, Zhejiang, China; ^2^Endoscopy Center, Zhejiang Xinan International Hospital, 314031 Jiaxing, Zhejiang, China

**Keywords:** digestive system neoplasms, perioperative period, anxiety, depression, postoperative complications, prognosis

## Abstract

**Background:**

To identify perioperative anxiety and depressive symptom trajectories in patients with digestive system malignancies undergoing surgery and to examine their associations with biomarkers and postoperative outcomes.

**Methods:**

This retrospective cohort study included 285 patients with digestive system malignancies who underwent elective surgery from January 2019 to December 2025 at Zhejiang Xinan International Hospital. Anxiety and depressive symptoms were assessed using repeated Hospital Anxiety and Depression Scale (HADS) total scores at T1 (1–3 days preoperatively), T2 (postoperative day 3), and T3 (postoperative day 7). Self-Rating Anxiety Scale, Perceived Stress Scale, Pittsburgh Sleep Quality Index, and Social Support Rating Scale scores were also extracted. Biomarkers, including cortisol, interleukin-6 (IL-6), tumor necrosis factor-α (TNF-α), C-reactive protein (CRP), and brain-derived neurotrophic factor (BDNF), were obtained from routine laboratory records. Latent class growth analysis was used to identify symptom trajectories. Multivariate logistic regression was performed to identify predictors of chronic trajectory membership.

**Results:**

Three trajectories were identified: resilient (42.1%), recovery (38.2%), and chronic (19.6%). Stage III patients had higher HADS scores than Stage I patients (18.6 ± 4.8 vs. 12.4 ± 3.6, *p* < 0.001). The chronic group showed higher postoperative IL-6 (8.2 ± 2.4 vs. 4.8 ± 1.6 pg/mL), TNF-α (18.6 ± 5.2 vs. 10.2 ± 3.4 pg/mL), and CRP levels, and lower BDNF levels than the resilient group. Chronic trajectory membership was associated with higher complication rates, longer hospital stay, higher 30-day readmission, and lower Quality of Recovery-15 (QoR-15) scores. Independent predictors included Stage III disease, psychiatric history, preoperative sleep disturbance, and reduced social support. The prediction model showed good (area under the receiver operating characteristic curve (AUC) = 0.856).

**Conclusions:**

Perioperative psychological distress follows distinct trajectories in patients with digestive system malignancies. Persistent distress is associated with inflammatory activation and poorer postoperative recovery, supporting early risk stratification and targeted psychological intervention.

## Introduction

Digestive system malignancies, including gastrointestinal tract cancers and hepatobiliary-pancreatic cancers, represent a major global health burden with significant morbidity and mortality [1, 2]. Surgical resection remains the cornerstone of curative treatment; however, the perioperative period constitutes a particularly vulnerable phase characterized by profound physical and psychological challenges [3, 4]. The diagnosis of cancer and prospect of major surgery create a perfect storm of psychological stressors that can profoundly influence both immediate surgical outcomes and long-term prognosis.

Patients with digestive system malignancies during the perioperative period face dual psychological stressors from cancer diagnosis and surgical trauma, and anxiety or depressive symptoms are frequently observed before surgery. Studies in preoperative colorectal cancer patients have reported relatively high Hospital Anxiety and Depression Scale (HADS)-detected anxiety and depressive symptoms, while studies in gastric cancer patients also suggest that preoperative anxiety and depression are clinically relevant concerns. However, reported prevalence varies according to tumor type, assessment timing, scale, and cutoff value [5, 6, 7]. Therefore, longitudinal assessment is needed to better characterize perioperative psychological symptom patterns in this population. These psychiatric symptoms not only severely impair patients’ quality of life but also influence surgical stress response, wound healing, and postoperative recovery through neuroendocrine-immune regulatory mechanisms [8, 9].

Perioperative anxiety and depressive symptoms are not static but demonstrate significant individual heterogeneity and temporal dynamics. Some patients exhibit remarkable psychological resilience and can rapidly adapt to stress, while others experience persistent psychological distress, potentially developing clinically significant anxiety disorders or depressive disorders [10, 11]. This heterogeneity suggests the existence of different symptom trajectory subgroups, and identifying these trajectory patterns is of great significance for precisely implementing psychiatric interventions.

The pathophysiology of surgical stress involves bidirectional communication between psychological states and physiological function through neuroendocrine and immune pathways [12, 13, 14]. Activation of the hypothalamic-pituitary-adrenal (HPA) axis leads to sustained cortisol elevation, while sympathetic activation increases catecholamine secretion. Pro-inflammatory cytokines such as interleukin-6 (IL-6) and tumor necrosis factor-α (TNF-α) participate in complex signaling cascades that influence wound healing, infection susceptibility, and potentially tumor biology [15, 16].

Accordingly, this study addressed two primary research questions. First, what distinct longitudinal trajectories of perioperative anxiety and depressive symptoms can be identified in patients with digestive system malignancies undergoing elective surgery, using repeated HADS total scores from the preoperative period to postoperative day 7? Second, are these trajectories associated with baseline clinical and psychosocial factors, neuroendocrine-inflammatory biomarkers, and postoperative recovery outcomes? Within this framework, we also explored whether routinely available preoperative variables could be used to construct a clinically feasible risk prediction model for identifying patients at high risk for persistent perioperative psychological distress.

## Methods

### Study Design and Setting

This retrospective longitudinal cohort study was conducted by reviewing the existing medical records of patients treated at Zhejiang Xinan International Hospital between January 2019 and December 2025. This study was approved by the Ethics Committee of Zhejiang Xinan International Hospital (Ethics Approval Number: 2026-003). Written informed consent had been obtained from all patients at the time of clinical management, and the present study retrospectively extracted relevant data from existing medical records. The study was conducted in accordance with the ethical principles of the Declaration of Helsinki and was reported according to Strengthening the Reporting of Observational Studies in Epidemiology (STROBE) guidelines for observational studies [17, 18].

### Inclusion and Exclusion Criteria

Inclusion criteria: (1) histopathologically confirmed digestive system malignancy, including gastrointestinal tract cancers (esophageal, gastric, and colorectal cancers) and hepatobiliary-pancreatic cancers (hepatic, biliary tract, and pancreatic cancers); (2) scheduled for first elective surgical resection with curative intent for the current malignancy; (3) age 18–75 years; (4) Eastern Cooperative Oncology Group (ECOG) performance status 0–2; (5) complete psychological assessment data available at all three predefined perioperative time points (T1, T2, and T3); (6) anticipated hospital stay ≥7 days; and (7) no prior treatment for the current malignancy except neoadjuvant therapy.

Exclusion criteria: (1) emergency surgery; (2) Stage IV disease with distant metastases; (3) current psychiatric medication use or active psychiatric/psychological treatment; (4) cognitive impairment, defined as Mini-Mental State Examination (MMSE) score <24; (5) American Society of Anesthesiologists physical status class IV or higher; (6) re-operation or secondary surgery for recurrent, residual, or previously treated digestive system malignancy; (7) concurrent malignancy; (8) incomplete key clinical, laboratory, operative, or follow-up records required for outcome assessment; and (9) withdrawal of consent or loss to follow-up before completion of postoperative outcome evaluation.

Patients undergoing re-operation or secondary oncologic surgery were excluded because such procedures may involve substantially different physiological stress responses, surgical risk profiles, and psychological burden compared with first elective curative resection. The ECOG performance status criterion was used to ensure adequate functional status for elective oncologic surgery, the MMSE threshold was used to exclude patients with possible cognitive impairment that could compromise self-report scale validity, and American Society of Anesthesiologists (ASA) class IV or higher was excluded to avoid enrolling patients with severe systemic disease and substantially different perioperative risk profiles. The ECOG scale is widely used in oncology trials, MMSE <24 is commonly used as a cognitive impairment threshold, and the ASA classification is widely used in perioperative risk assessment [19, 20, 21].

### Data Collection and Psychological Assessment

All demographic, clinical, psychological, and laboratory data were retrospectively extracted from the electronic medical records and hospital databases of Zhejiang Xinan International Hospital. Demographic variables included age, sex, education level, marital status, and employment status. Clinical variables included tumor type, tumor-node-metastasis (TNM) stage, neoadjuvant therapy, surgical procedure, ASA classification, and surgical complexity. Surgical complexity was categorized based on operative records, with high surgical complexity defined as procedures involving combined organ resection, multivisceral resection, extended lymphadenectomy, or prolonged operative time according to institutional surgical documentation.

As part of routine perioperative clinical care at our institution, psychological screening data were documented in the medical records at the corresponding perioperative time points. The Chinese versions of the HADS, Self-Rating Anxiety Scale (SAS), and Perceived Stress Scale (PSS-10) were used in routine assessment. In routine practice, these questionnaires were generally administered as standardized self-report instruments under the guidance of trained nursing or clinical staff using uniform scoring instructions. However, because this study was based on retrospective chart review, we were unable to fully verify whether all assessments had been completed under completely identical research-level conditions across all patients and time points.

(1) HADS: Documented HADS records in the medical charts were reviewed. The HADS comprises an anxiety subscale (HADS-A) and a depression subscale (HADS-D), each containing 7 items scored 0–3. HADS-A and HADS-D scores each range from 0 to 21, and the HADS total score is the sum of both subscales, ranging from 0 to 42. In this study, a HADS total score ≥15 was used to indicate clinically significant psychological distress or an increased need for psychosocial care, based on prior cancer-related distress screening research. Higher HADS total scores were interpreted as reflecting greater anxiety and depressive symptom burden. The HADS was originally developed by Zigmond and Snaith and has been used for cancer-related psychological distress screening [22, 23].

(2) SAS: Documented SAS results were extracted from the medical records as an auxiliary measure of anxiety symptom severity. The SAS contains 20 items, each scored from 1 to 4. The raw score ranges from 20 to 80 and is converted to a standard score by multiplying by 1.25, yielding a standard score range of 25 to 100. Higher scores indicate more severe anxiety symptoms. In the present study, SAS standard scores were analyzed as continuous variables rather than being used to define diagnostic categories. The SAS was originally developed by Zung and is widely used for anxiety symptom screening [24].

(3) PSS-10: Documented PSS-10 results were extracted from the medical records to reflect perceived stress over the past month. The PSS-10 consists of 10 items scored from 0 to 4, with total scores ranging from 0 to 40. Higher scores indicate greater perceived stress. No diagnostic cutoff was applied in this study; PSS-10 was analyzed as a continuous measure. The Chinese version of PSS-10 has demonstrated acceptable reliability and validity in prior validation studies [25, 26].

(4) Pittsburgh Sleep Quality Index (PSQI): Documented PSQI results were extracted from the medical records to assess sleep quality. The PSQI consists of seven component scores, each ranging from 0 to 3, with a total score ranging from 0 to 21. Higher scores indicate poorer sleep quality. In this study, a PSQI total score >7, equivalent to a global score ≥8, was used to define PSQI-defined preoperative sleep disturbance rather than a formal clinical diagnosis of insomnia. This cutoff was selected based on prior validation evidence in cancer patients, in which a PSQI global score of 8 showed the best sensitivity and specificity for identifying sleep disturbance. The Chinese/Taiwanese version of the PSQI has been reported to be reliable and valid for assessing subjective sleep quality in cancer populations [27, 28].

(5) Social Support Rating Scale (SSRS): Documented SSRS results were extracted from the medical records to assess social support. The Chinese SSRS is a 10-item instrument covering three dimensions: objective support, subjective support, and support utilization. Total SSRS scores range from 12 to 66, with higher scores indicating stronger social support [29]. In the present study, SSRS was analyzed both as a continuous variable and as a categorical variable for risk stratification. Because there is no universally accepted cutoff for defining low social support using the SSRS in perioperative patients with digestive system malignancies, an SSRS total score <30 was used as a prespecified operational threshold to identify patients with relatively reduced social support in this cohort. This threshold was not intended to represent a validated diagnostic cutoff. The distribution of SSRS scores across trajectory groups is additionally presented in the Results section and **Supplementary Table 5**.

(6) History of Psychiatric Disorders: This variable was identified through retrospective review of medical records and prior medical history documentation. History of psychiatric disorders was defined as patient self-report or documented medical records of previous diagnoses of anxiety disorders, depressive disorders, or other psychiatric disorders, including patients who had received treatment, pharmacological or psychological, but had discontinued treatment for at least 3 months. Patients currently receiving psychiatric treatment were excluded. Recorded psychiatric histories mainly included generalized anxiety disorder, panic disorder, social anxiety disorder, major depressive disorder, and persistent depressive disorder, based on previous clinical diagnoses in medical records or patient-reported confirmed diagnoses.

Because the retrospective electronic database retained total and subscale scores rather than complete item-level responses for some scales, internal consistency coefficients could not be recalculated for all instruments in the present cohort. Therefore, the psychometric basis of the scales was supported by prior validation literature.

### Biomarker Measurements

Laboratory records of fasting venous blood test results at T1 (1–3 days preoperatively) and T3 (postoperative day 7) were retrospectively extracted from the hospital database. Recorded serum cortisol results were obtained from electrochemiluminescence immunoassay, whereas IL-6, TNF-α, C-reactive protein (CRP), and brain-derived neurotrophic factor (BDNF) were obtained from the corresponding laboratory testing records in the hospital database [30]. All biomarker data were derived from routine clinical testing performed by the hospital laboratory according to standard institutional procedures.

For the main between-group comparison, postoperative biomarker values at T3 were emphasized because they more directly reflected postoperative neuroendocrine-inflammatory status in relation to perioperative symptom trajectories. Biomarker values at both T1 and T3 were additionally summarized to describe temporal changes. Correlation analyses were performed to examine the associations between HADS total scores and biomarker levels at T1 and T3, using Pearson or Spearman methods as appropriate according to data distribution.

### Clinical Outcome Measures

The primary psychological outcome of this study was the longitudinal trajectory pattern of perioperative anxiety and depressive symptoms, operationalized as changes in HADS total scores across three assessment time points: T1, 1–3 days preoperatively; T2, postoperative day 3; and T3, postoperative day 7. Thus, the primary psychological outcome reflected the temporal pattern and rate of change in symptom scores rather than a single incidence endpoint. Baseline clinically significant psychological distress was defined as a HADS total score ≥15. HADS-A, HADS-D, SAS, and PSS-10 scores were analyzed as additional measures of symptom severity and perceived stress.

The primary clinical outcome was postoperative complications, identified from inpatient medical records and classified according to the Clavien-Dindo system, with Grade II or higher defined as clinically relevant complications. The Clavien-Dindo classification is widely used to grade postoperative complication severity, and Grade II indicates complications requiring pharmacological treatment beyond that allowed for Grade I [31]. In addition to the overall complication rate, postoperative complications were further categorized by type based on the medical records, including infectious complications, anastomotic leakage, gastrointestinal dysfunction, pulmonary complications, postoperative bleeding, urinary complications, and cardiovascular complications. Because a single patient could experience more than one postoperative complication, these complication categories were not mutually exclusive.

For severity analysis, the highest recorded Clavien-Dindo grade per patient was used. Secondary clinical outcomes included length of hospital stay, intensive care unit (ICU) stay duration, 30-day readmission rate, and postoperative recovery quality assessed using the Quality of Recovery-15 (QoR-15). The QoR-15 is a 15-item patient-reported measure of postoperative recovery quality, with total scores ranging from 0 to 150 and higher scores indicating better recovery quality. The QoR-15 was developed and psychometrically evaluated as a concise postoperative recovery measure [32].

### Statistical Analysis

Statistical analyses were performed using SPSS 26.0 (IBM Corp., Armonk, NY, USA) and Mplus 8.3 (Muthén & Muthén, Los Angeles, CA, USA). Latent class growth analysis (LCGA) was performed using Mplus 8.3 to identify distinct trajectories of perioperative anxiety and depressive symptoms based on repeated HADS total scores across T1, T2, and T3. Models with increasing numbers of latent classes were estimated sequentially, beginning with a 1-class model and continuing until model fit no longer improved meaningfully or additional classes became clinically uninterpretable. Model selection was based on a combination of statistical fit indices, including Akaike Information Criterion (AIC), Bayesian Information Criterion (BIC), sample-size adjusted BIC (aBIC), entropy, the Lo-Mendell-Rubin adjusted likelihood ratio test (LMR-LRT), and the bootstrap likelihood ratio test (BLRT), together with class size and clinical interpretability. Lower AIC, BIC, and aBIC values indicated better model fit, while higher entropy indicated better classification accuracy.

After the optimal unconditional class solution was selected, age and sex were entered as covariates in a conditional LCGA model to assess whether the trajectory classification remained robust after adjustment for key demographic factors. The age- and sex-adjusted estimated trajectories were used for graphical presentation. Trajectory groups were labeled post hoc according to the estimated HADS total score patterns and clinically meaningful thresholds: the resilient trajectory reflected persistently low symptom scores, the recovery trajectory reflected initially elevated symptoms with subsequent improvement, and the chronic trajectory reflected persistently high symptom burden across all three time points. Because this was a retrospective observational study, no a priori power analysis specific to LCGA was performed; therefore, model adequacy was judged based on total sample size, class proportions, entropy, posterior classification quality, and clinical interpretability.

Normality of continuous variables was assessed using the Shapiro-Wilk test and visual inspection of histograms and Q-Q plots. Homogeneity of variance was evaluated using Levene’s test. Normally distributed continuous variables were presented as mean ± standard deviation and compared using one-way analysis of variance (ANOVA). Non-normally distributed variables were presented as median and interquartile range and compared using the Kruskal-Wallis test. For two-group comparisons, Student’s t-test or Mann-Whitney U test was used as appropriate. Categorical variables were presented as frequency and percentage and compared using χ^2^ test or Fisher’s exact test, as appropriate.

To construct a risk prediction model for chronic trajectory membership, univariate analyses were first conducted comparing all baseline variables between the chronic trajectory group and the non-chronic trajectory group. Variables with *p* < 0.10 in univariate analyses were considered candidate predictors. In addition, clinically relevant covariates, including age, sex, neoadjuvant therapy, and surgical complexity, were entered into the multivariate logistic regression model regardless of univariate significance to reduce potential confounding. Before multivariate logistic regression, multicollinearity among candidate predictors was assessed using variance inflation factors (VIFs) and tolerance values. A VIF <5 and tolerance >0.2 were considered indicative of the absence of substantial multicollinearity. Model performance was assessed by the area under the receiver operating characteristic curve (AUC). All tests were two-sided, with *p* < 0.05 considered statistically significant.

## Results

### Baseline Characteristics

A total of 285 patients were included in the analysis. Baseline demographic, clinical, psychosocial, and preoperative biomarker characteristics across the three trajectory groups are shown in Table 1. Age, sex, education level, marital status, employment status, tumor type, ASA classification, and surgical approach were not significantly different across the resilient, recovery, and chronic trajectory groups. However, patients in the chronic trajectory group had a higher proportion of Stage III disease, psychiatric history, preoperative sleep disturbance, reduced social support, neoadjuvant therapy, and high surgical complexity. In addition, preoperative inflammatory markers, including IL-6, TNF-α, and CRP, were higher in the chronic trajectory group, whereas BDNF levels were lower. These baseline differences were considered in subsequent multivariate analyses.

**Table 1.** **Baseline characteristics and preoperative biomarkers across trajectory groups (N = 285)**.

Variable		Resilient (n = 120)	Recovery (n = 109)	Chronic (n = 56)	F/χ^2^	*p*
Age (years, $\bar{x}$ ± s)		57.8 ± 10.1	59.6 ± 10.3	61.4 ± 10.8	2.11	0.123
Sex, n (%)					0.24	0.886
	Male	73 (60.8)	64 (58.7)	35 (62.5)		
	Female	47 (39.2)	45 (41.3)	21 (37.5)		
Education level, n (%)					2.48	0.290
	<High school	47 (39.2)	47 (43.1)	29 (51.8)		
	≥High school	73 (60.8)	62 (56.9)	27 (48.2)		
Marital status, n (%)					1.02	0.600
	Married	107 (89.2)	94 (86.2)	47 (83.9)		
	Unmarried/Divorced/Widowed	13 (10.8)	15 (13.8)	9 (16.1)		
Employment status, n (%)					1.95	0.377
	Employed	71 (59.2)	60 (55.0)	27 (48.2)		
	Unemployed/Retired	49 (40.8)	49 (45.0)	29 (51.8)		
Tumor type, n (%)					1.05	0.983
	Gastric cancer	40 (33.3)	41 (37.6)	21 (37.5)		
	Colorectal cancer	59 (49.2)	50 (45.9)	27 (48.2)		
	Esophageal cancer	13 (10.8)	11 (10.1)	4 (7.1)		
	Hepatobiliary-pancreatic cancer	8 (6.7)	7 (6.4)	4 (7.1)		
Tumor stage, n (%)					20.73	<0.001
	Stage I	33 (27.5)	20 (18.3)	5 (8.9)		
	Stage II	52 (43.3)	36 (33.0)	16 (28.6)		
	Stage III	35 (29.2)	53 (48.6)	35 (62.5)		
ASA classification, n (%)					2.95	0.566
	ASA I	21 (17.5)	17 (15.6)	7 (12.5)		
	ASA II	76 (63.3)	68 (62.4)	32 (57.1)		
	ASA III	23 (19.2)	24 (22.0)	17 (30.4)		
Surgical approach, n (%)					1.24	0.538
	Open surgery	37 (30.8)	37 (33.9)	22 (39.3)		
	Minimally invasive surgery	83 (69.2)	72 (66.1)	34 (60.7)		
Psychiatric history, n (%)					54.26	<0.001
	Yes	4 (3.3)	10 (9.2)	24 (42.9)		
	No	116 (96.7)	99 (90.8)	32 (57.1)		
Preoperative sleep disturbance, n (%)					36.62	<0.001
	Yes, PSQI >7	16 (13.3)	38 (34.9)	32 (57.1)		
	No, PSQI ≤7	104 (86.7)	71 (65.1)	24 (42.9)		
Reduced social support, n (%)					27.12	<0.001
	Yes, SSRS <30	16 (13.3)	34 (31.2)	28 (50.0)		
	No, SSRS ≥30	104 (86.7)	75 (68.8)	28 (50.0)		
Neoadjuvant therapy, n (%)					6.39	0.041
	Yes	31 (25.8)	38 (34.9)	25 (44.6)		
	No	89 (74.2)	71 (65.1)	31 (55.4)		
Surgical complexity, n (%)					14.84	0.001
	High	24 (20.0)	31 (28.4)	27 (48.2)		
	Low/Moderate	96 (80.0)	78 (71.6)	29 (51.8)		
Cortisol at T1 (nmol/L)		298 ± 54	336 ± 62	382 ± 70	15.84	<0.001
IL-6 at T1 (pg/mL)		3.6 ± 1.2	4.4 ± 1.4	5.3 ± 1.8	12.46	<0.001
TNF-α at T1 (pg/mL)		8.4 ± 2.8	10.6 ± 3.2	12.8 ± 3.8	14.26	<0.001
CRP at T1 (mg/L)		9.6 ± 3.2	12.4 ± 4.0	15.8 ± 5.4	13.82	<0.001
BDNF at T1 (ng/mL)		14.2 ± 4.0	11.8 ± 3.6	9.2 ± 3.0	11.94	<0.001

Note: ASA, American Society of Anesthesiologists; PSQI, Pittsburgh Sleep Quality Index; SSRS, Social Support Rating Scale; IL-6, interleukin-6; TNF-α, tumor necrosis factor-α; CRP, C-reactive protein; BDNF, brain-derived neurotrophic factor.

### Stress Trajectory Patterns

To determine the optimal trajectory solution, models with 1 to 4 latent classes were compared. As shown in Table 2, the 3-class model demonstrated lower AIC, BIC, and adjusted BIC values than the 1-class and 2-class models, with high entropy (0.89), significant LMR-LRT and BLRT results compared with the 2-class model, and clinically interpretable class structures. Although the 4-class model showed a slightly lower AIC, it had a higher BIC, lower entropy, and an additional small subgroup, which reduced model parsimony and clinical interpretability.

**Table 2.** **Fit indices for latent class growth models**.

Model	AIC	aBIC	BIC	Entropy	LMR-LRT P	BLRT P	Class sizes (%)
1-class	4912.8	4918.6	4946.9	—	—	—	100.0
2-class	4868.4	4876.1	4908.7	0.82	<0.001	<0.001	61.4 / 38.6
3-class	4807.6	4816.8	4856.3	0.89	0.021	<0.001	42.1 / 38.2 / 19.6
4-class	4803.9	4814.7	4862.0	0.84	0.187	0.064	40.7 / 33.0 / 20.0 / 6.3

Note: AIC, Akaike Information Criterion; aBIC, sample-size adjusted Bayesian Information Criterion; BIC, Bayesian Information Criterion; LMR-LRT, Lo-Mendell-Rubin adjusted likelihood ratio test; BLRT, bootstrap likelihood ratio test.

**Fig. 1.**
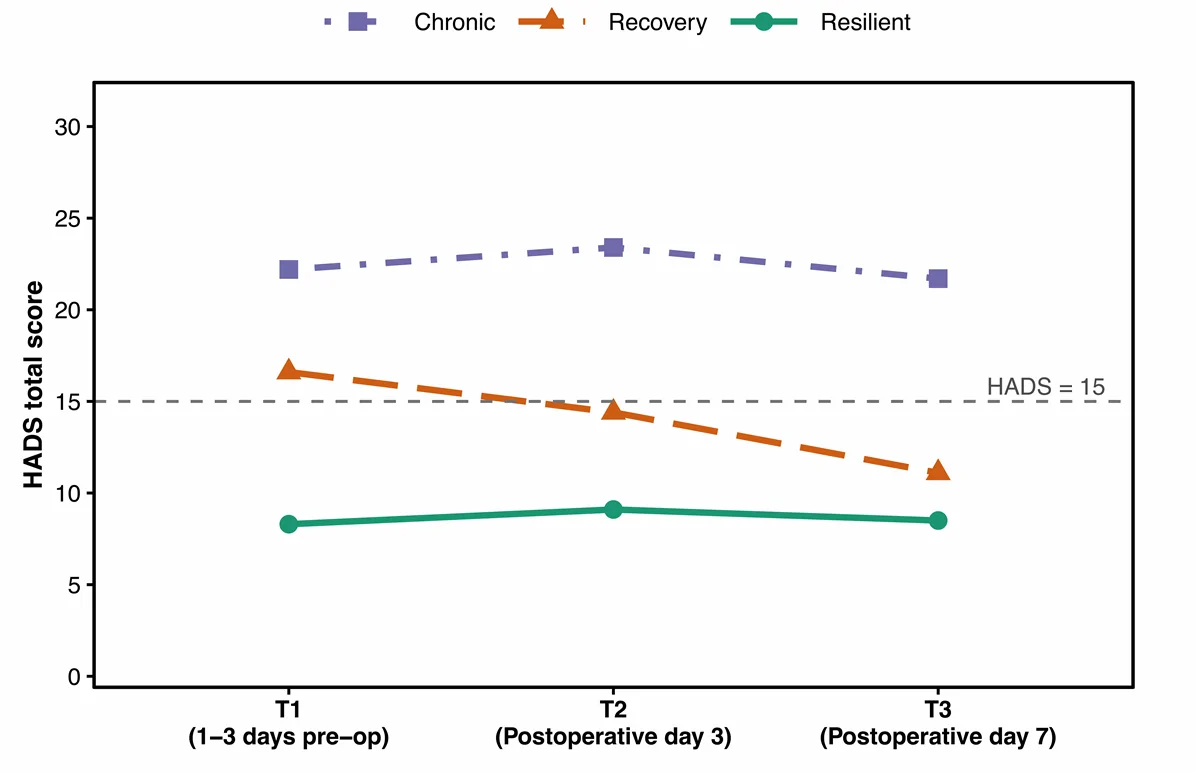
**Age- and sex-adjusted trajectories of perioperative anxiety and depressive symptoms identified by latent class growth analysis**. The resilient group showed persistently low HADS total scores across the three perioperative time points, the recovery group showed initially elevated symptoms with gradual improvement, and the chronic group showed persistently high symptom burden throughout follow-up. The dashed horizontal line indicates a HADS total score of 15, which was used to define clinically significant psychological distress. HADS, Hospital Anxiety and Depression Scale; T1, 1–3 days preoperatively; T2, postoperative day 3; T3, postoperative day 7.

The selected 3-class solution remained stable after adjustment for age and sex in the conditional LCGA model. The age- and sex-adjusted estimated trajectories are shown in Fig. 1. Three distinct patterns were identified: a resilient trajectory, characterized by persistently low HADS total scores; a recovery trajectory, characterized by initially elevated symptoms followed by gradual improvement; and a chronic trajectory, characterized by persistently high symptom burden across all three perioperative time points. The smallest subgroup was the chronic trajectory group, comprising 56 patients (19.6%), which supported the relative stability of the final classification despite the absence of a formal a priori power analysis for LCGA.

### Symptom Severity Across Tumor Stages

Symptom severity demonstrated significant tumor stage specificity (Table 3). Stage III patients showed the highest HADS total scores (18.6 ± 4.8), which were significantly higher than those of Stage I patients (12.4 ± 3.6, *p* < 0.001) and Stage II patients (15.8 ± 4.2, *p* < 0.01). Similar stage-related differences were observed for HADS-A, HADS-D, SAS standard scores, and PSS-10 scores. The proportion of patients in the chronic trajectory increased numerically with tumor stage, from 8.6% in Stage I to 15.4% in Stage II and 28.5% in Stage III; the overall distribution differed significantly across tumor stages (χ^2^ = 14.82, *p* < 0.001).

**Table 3.** **Psychological stress levels by tumor stage**.

Variable	Stage I (n = 58)	Stage II (n = 104)	Stage III (n = 123)	F/χ²	*p*
HADS Total	12.4 ± 3.6	15.8 ± 4.2	18.6 ± 4.8***^#^	12.46	<0.001
HADS-A	6.8 ± 2.4	8.6 ± 3.2	10.2 ± 3.8***	9.86	<0.001
HADS-D	5.6 ± 2.2	7.2 ± 2.8	8.4 ± 3.4**	6.42	0.002
SAS standard score	45.2 ± 8.6	51.8 ± 9.4	57.6 ± 10.2***^#^	13.06	<0.001
PSS-10	14.2 ± 4.6	18.6 ± 5.4	22.4 ± 6.2***^#^	14.28	<0.001
Chronic trajectory, n (%)	5 (8.6)	16 (15.4)	35 (28.5)	14.82^†^	<0.001

Note: HADS, Hospital Anxiety and Depression Scale; HADS-A, HADS anxiety subscale; HADS-D, HADS depression subscale; SAS, Self-Rating Anxiety Scale; PSS-10, Perceived Stress Scale-10.**p < 0.01,***p < 0.001 vs. Stage I; ^#^*p* < 0.01 vs. Stage II; ^†^χ^2^ test for overall comparison among the three tumor stages.

### Stress Trajectories and Neuroendocrine-Immune Markers

Patients in the chronic trajectory demonstrated significant neuroendocrine-immune activation. Biomarker values at both T1 and T3 are summarized in **Supplementary Table 1**. At baseline (T1), patients in the chronic trajectory already showed higher levels of cortisol, IL-6, TNF-α, and CRP, as well as lower BDNF levels, than those in the resilient trajectory. These between-group differences became more pronounced at T3, suggesting persistent postoperative biological dysregulation in the chronic symptom group. Because T3 values more directly reflected postoperative biological status in relation to perioperative symptom trajectories, the between-group comparison at T3 is presented in Table 4. At T3, serum cortisol levels were significantly higher in the chronic group than in the resilient group (468 ± 86 vs. 326 ± 62 nmol/L, *p* < 0.001). Similarly, inflammatory markers were significantly elevated in the chronic group, including IL-6 (8.2 ± 2.4 vs. 4.8 ± 1.6 pg/mL, *p* < 0.001), TNF-α (18.6 ± 5.2 vs. 10.2 ± 3.4 pg/mL, *p* < 0.001), and CRP (42.6 ± 12.8 vs. 28.4 ± 8.6 mg/L, *p* < 0.001). In contrast, BDNF levels were markedly lower in the chronic group than in the resilient group (6.8 ± 2.4 vs. 12.6 ± 3.8 ng/mL, *p* < 0.001).

**Table 4.** **Biomarker levels across trajectory groups at T3**.

Biomarker	Resilient (n = 120)	Recovery (n = 109)	Chronic (n = 56)	F	*p*
Cortisol (nmol/L)	326 ± 62	398 ± 74	468 ± 86***	24.68	<0.001
IL-6 (pg/mL)	4.8 ± 1.6	6.4 ± 2.0	8.2 ± 2.4***	28.42	<0.001
TNF-α (pg/mL)	10.2 ± 3.4	14.2 ± 4.2	18.6 ± 5.2***	22.16	<0.001
CRP (mg/L)	28.4 ± 8.6	35.2 ± 10.4	42.6 ± 12.8***	18.62	<0.001
BDNF (ng/mL)	12.6 ± 3.8	9.4 ± 3.2	6.8 ± 2.4***	16.84	<0.001

Note: IL-6, interleukin-6; TNF-α, tumor necrosis factor-α; CRP, C-reactive protein; BDNF, brain-derived neurotrophic factor. ***p < 0.001 vs. Resilient group.

Correlation analyses between HADS total scores and biomarker levels at T1 and T3 are presented in **Supplementary Table 2**. Higher HADS total scores were moderately correlated with higher postoperative cortisol, IL-6, TNF-α, and CRP levels, and inversely correlated with BDNF levels. These findings indicate that persistent perioperative psychological distress was associated with, rather than proven to mediate, postoperative neuroendocrine-immune dysregulation.

### Impact of Stress Trajectories on Postoperative Outcomes

The chronic trajectory was associated with significantly worse postoperative outcomes (Table 5). The rate of overall postoperative complications was highest in the chronic group, followed by the recovery group and the resilient group (38.9% vs. 21.1% vs. 14.2%, *p* < 0.001). Patients in the chronic trajectory also had a longer length of hospital stay (14.2 ± 4.6 vs. 11.4 ± 3.6 vs. 9.2 ± 2.8 days, *p* < 0.001), a higher 30-day readmission rate (16.1% vs. 8.3% vs. 5.0%, *p* = 0.024), and lower quality of recovery (QoR-15 score) (102.6 ± 20.8 vs. 118.2 ± 16.4 vs. 128.4 ± 12.6, *p* < 0.001), indicating poorer postoperative recovery quality.

**Table 5.** **Postoperative outcomes across trajectory groups**.

Outcome	Resilient (n = 120)	Recovery (n = 109)	Chronic (n = 56)	F/χ^2^	*p*
Overall postoperative complications, n (%)	14.2	21.1	38.9***	14.26	<0.001
Infectious complications, n (%)	7.5	12.8	23.2**	10.28	0.006
Length of hospital stay (days, $\bar{x}$ ± s)	9.2 ± 2.8	11.4 ± 3.6	14.2 ± 4.6***	18.64	<0.001
30-day readmission, n (%)	5.0	8.3	16.1*	7.42	0.024
Quality of recovery (QoR-15 score, $\bar{x}$ ± s)	128.4 ± 12.6	118.2 ± 16.4	102.6 ± 20.8***	24.82	<0.001

Note: QoR-15, Quality of Recovery-15. Higher QoR-15 scores indicate better postoperative recovery quality. ***p < 0.001, **p < 0.01, *p < 0.05 vs. Resilient group.

To further characterize the postoperative recovery profile, the distribution of specific complication types across trajectory groups is summarized in **Supplementary Table 3**. Infectious complications were the most common postoperative adverse events, followed by gastrointestinal dysfunction and pulmonary complications. These complication categories were observed more frequently in the chronic trajectory group than in the resilient group, suggesting that persistent perioperative psychological distress may be associated with broader impairment in postoperative recovery rather than with a single isolated complication type.

The distribution of complication severity is shown in **Supplementary Table 4**. Among patients who developed postoperative complications, those in the chronic trajectory group had a higher proportion of Grade III–IV complications, whereas most complications in the resilient and recovery groups were Grade II. This finding suggests that chronic perioperative distress may be associated not only with a higher complication burden but also with greater complication severity.

### Risk Prediction Model for Chronic Stress Trajectory

In addition to the binary definition of reduced social support (SSRS <30) used in the regression analysis, SSRS total scores were also compared across trajectory groups. Patients in the chronic trajectory group had the lowest SSRS scores, followed by the recovery group, whereas the resilient group had the highest scores. The full distribution of SSRS total scores and the proportion of patients with reduced social support in each trajectory group are shown in **Supplementary Table 5**. These findings further support the association between reduced social support and persistent perioperative psychological distress.

Univariate analyses showed significant differences between the chronic and non-chronic groups (*p* < 0.10) for tumor stage, history of psychiatric disorders, preoperative sleep disturbance, reduced social support (operationally defined as SSRS <30), and neoadjuvant therapy. Age, sex, neoadjuvant therapy, and surgical complexity were additionally entered into the multivariate model as clinically relevant potential confounders. Before multivariate logistic regression, multicollinearity diagnostics showed no evidence of substantial collinearity among the candidate predictors, including psychiatric history and preoperative sleep disturbance, and all variables met the predefined criteria for inclusion in the final model.

After adjustment for age, sex, neoadjuvant therapy, and surgical complexity, multivariate logistic regression identified four independent predictors of chronic trajectory membership (Table 6): advanced tumor stage (Stage III vs. I-II, odds ratio (OR) = 2.94, 95% confidence interval (CI): 1.68–5.14), history of psychiatric disorders (OR = 3.86, 95% CI: 1.96–7.62), preoperative sleep disturbance (OR = 2.48, 95% CI: 1.42–4.34), and reduced social support (OR = 2.26, 95% CI: 1.32–3.88). In contrast, age, sex, neoadjuvant therapy, and surgical complexity were not independently associated with chronic trajectory membership in the final adjusted model.

**Table 6.** **Multivariate logistic regression analysis for chronic trajectory prediction**.

Variable	B	SE	OR (95% CI)	Wald	*p*
Tumor stage III	1.078	0.286	2.94 (1.68–5.14)	14.22	<0.001
Psychiatric history	1.350	0.348	3.86 (1.96–7.62)	15.06	<0.001
Preoperative sleep disturbance	0.908	0.284	2.48 (1.42–4.34)	10.24	0.001
Reduced social support	0.816	0.276	2.26 (1.32–3.88)	8.74	0.003
Age	0.011	0.013	1.01 (0.99–1.04)	0.72	0.396
Male sex	0.184	0.267	1.20 (0.71–2.04)	0.48	0.488
Neoadjuvant therapy	0.228	0.291	1.26 (0.71–2.22)	0.61	0.434
Surgical complexity	0.307	0.298	1.36 (0.76–2.43)	1.06	0.304

Note: OR, odds ratio; CI, confidence interval; SE, standard error.

The risk prediction model based on these four predictors demonstrated excellent discriminative ability, with an AUC of 0.856 (95% CI: 0.812–0.900), sensitivity of 82.1%, specificity of 78.6%, positive predictive value of 64.3%, and negative predictive value of 90.2%. This model may be useful for preoperative screening to identify high-risk patients who may benefit from early psychiatric consultation and multidisciplinary psychological intervention.

## Discussion

This study systematically delineated the dynamic trajectories of perioperative anxiety and depressive symptoms in patients with digestive system malignancies, identified three distinct trajectory patterns, and established a risk prediction model based on psychiatric assessment. Approximately one-fifth of patients exhibited a chronic high anxiety and depressive symptom trajectory, which was closely associated with persistent neuroendocrine-immune activation, higher risk of postoperative complications, and poorer clinical outcomes. Importantly, although no formal a priori power analysis for LCGA was performed, the final 3-class solution showed good entropy and a smallest class proportion of 19.6%, supporting the relative stability and interpretability of the trajectory classification in this cohort.

Previous studies predominantly used cross-sectional designs to assess the psychiatric and psychological status of cancer patients, neglecting the temporal dynamics and individual heterogeneity of symptoms [33, 34]. Most notably, the chronic trajectory (19.6%) represents patients with persistently elevated HADS total scores throughout the perioperative period. This persistent psychological distress not only severely impacts patients’ quality of life but may also indicate underlying clinical anxiety disorders or depressive disorders. Notably, 42.9% of patients in this group had a history of psychiatric disorders, significantly higher than the other two groups. History of psychiatric disorders is an important factor predicting the persistence of perioperative psychiatric symptoms [35], suggesting these patients may have dispositional vulnerability and are more prone to relapse or exacerbation of psychiatric symptoms when facing major stressors. In addition, advanced tumor stage was more frequently observed in the chronic trajectory group, consistent with prior evidence that tumor stage may be associated with preoperative psychological distress in gastric and colorectal cancer surgery populations [10].

The biological findings further support the clinical relevance of the chronic trajectory. Patients in this group demonstrated persistent HPA axis activation, reflected by elevated cortisol levels, together with a pro-inflammatory profile characterized by higher IL-6, TNF-α, and CRP levels. These observations are in line with the theoretical framework of psychoneuroimmunology, which proposes bidirectional interactions between chronic psychological stress, neuroendocrine dysregulation, and inflammatory activation [36, 37, 38]. Chronic anxiety and depressive states lead to HPA axis negative feedback dysregulation and decreased glucocorticoid receptor sensitivity, resulting in disrupted cortisol circadian rhythm and elevated baseline levels [38]. At the same time, these findings should be interpreted as associative rather than causal, because the retrospective design and temporal overlap between trajectory classification, biomarker measurements, and postoperative outcomes do not allow formal mediation inference.

These adverse psychological and biological profiles were paralleled by worse postoperative recovery. The chronic trajectory group had the highest postoperative complication rate, exceeding both the resilient and recovery groups, and also showed longer hospitalization, higher readmission, and poorer recovery quality. This pattern is broadly consistent with prior studies suggesting that psychological distress may adversely affect surgical outcomes [39, 40]. Several mechanisms may plausibly underlie this relationship, including neuroendocrine-immune dysregulation affecting wound healing and infection defense, reduced postoperative compliance and self-management, and the negative effect of sleep disturbance on tissue repair and recovery [41]. Moreover, the chronic trajectory group not only had a higher overall complication burden, but also showed a broader distribution of complication types and a higher proportion of more severe complications, suggesting that persistent perioperative distress may adversely affect multiple dimensions of surgical recovery rather than a single isolated endpoint.

The multivariable analysis further strengthened the clinical implications of these findings. History of psychiatric disorders remained the strongest independent predictor of chronic trajectory membership. It is important to emphasize that this variable referred to patients with previous confirmed diagnoses of anxiety disorders, depressive disorders, or other psychiatric disorders, including those who had received treatment but had discontinued it at least 3 months before surgery; thus, it represented psychiatric vulnerability rather than active psychiatric illness. This is clinically meaningful because psychiatric disorders often follow a relapsing course, and even patients in remission may experience recurrence when exposed to major life stressors such as cancer diagnosis and surgery [42]. In the present study, this association remained significant even after adjustment for age, sex, neoadjuvant therapy, and surgical complexity, supporting the relative independence of psychiatric history as a risk factor for persistent perioperative distress. Advanced tumor stage, preoperative sleep disturbance, and reduced social support were also independently associated with chronic distress, indicating that persistent perioperative psychological burden is shaped by the combined effects of disease severity, prior psychiatric susceptibility, sleep disturbance, and psychosocial resources.

Taken together, these findings support the potential clinical utility of the proposed risk prediction model. Because the four predictors included in the final model are all readily obtainable in routine perioperative assessment, the model may provide a practical basis for early risk stratification. On this basis, a feasible stepwise intervention strategy may be considered for high-risk patients. This could include preoperative screening with HADS, PSQI, and SSRS 1–3 days before surgery; early psychiatric or psychological consultation for patients identified as high risk; brief psychoeducation, supportive counseling, and sleep-focused behavioral guidance during hospitalization; reassessment at postoperative day 3 and postoperative day 7 for those with persistent symptoms; and telephone- or internet-based follow-up after discharge to maintain continuity of psychological support. Such an approach may enhance the practical value of the model by directly linking risk identification to actionable perioperative management.

This study has several limitations. First, as a retrospective single-center study, it is inherently subject to selection bias, and data extraction relied on electronic medical records, which may have contained incomplete, missing, or inconsistently documented information. Second, although we observed significant associations between chronic perioperative distress, neuroendocrine-inflammatory activation, and adverse postoperative outcomes, the observational design precludes causal inference. In particular, we cannot exclude the possibility that pre-existing biological dysregulation may have contributed to both persistent psychological distress and poor postoperative outcomes. Third, psychological symptoms were assessed using documented scale-based measures rather than standardized psychiatric interviews, which limited diagnostic precision. Fourth, although biomarker data were available at both T1 and T3, the temporal overlap between symptom trajectory classification, postoperative biomarker measurements, and postoperative outcomes limited our ability to formally test mediation pathways or infer causal biological mechanisms. Fifth, no a priori power analysis specific to LCGA was performed, and although the chronic trajectory subgroup remained above commonly used minimum class-size thresholds, its relatively small size may have reduced the precision of some subgroup analyses, particularly for biomarker and postoperative outcome comparisons. Finally, the exclusion of patients receiving active psychiatric treatment may have introduced additional selection bias and may limit the generalizability of our findings. In addition, although psychological scale data were routinely documented in the medical records, the retrospective nature of the study did not allow full verification that all assessments were administered under completely standardized conditions across different staff and time points, which may have introduced measurement variability. Moreover, although the SSRS has been widely used and has shown acceptable psychometric performance in Chinese populations, including oncology-related studies, evidence specifically in perioperative digestive system malignancies surgical cohorts remains limited. In the present study, the SSRS total score <30 threshold was used as a prespecified operational cutoff for risk stratification rather than as a universally validated diagnostic criterion for low social support. Therefore, the findings related to relatively reduced social support should be interpreted cautiously, and future prospective studies are needed to validate the optimal SSRS cutoff in perioperative patients with digestive system malignancies.

## Conclusions

Perioperative anxiety and depressive symptoms in patients with digestive system malignancies followed three distinct longitudinal trajectories: resilient, recovery, and chronic. The chronic trajectory, observed in approximately one-fifth of patients, was characterized by persistently elevated HADS total scores and was associated with neuroendocrine-inflammatory activation, higher complication burden, longer hospitalization, higher readmission, and poorer postoperative recovery quality. Advanced tumor stage, psychiatric history, preoperative sleep disturbance, and reduced social support independently predicted chronic trajectory membership. These findings suggest that perioperative psychological distress should be dynamically monitored rather than assessed only once before surgery. A risk model based on routinely available clinical and psychosocial variables may support early identification of high-risk patients and facilitate timely psychiatric consultation, sleep management, social support enhancement, and multidisciplinary perioperative psychological care.

## Availability of Data and Materials

The datasets used and/or analysed during the current study were available from the corresponding author on reasonable request.

## Supplementary Material

Supplementary material associated with this article can be found, in the online version, at https://doi.org/10.62641/aep.v54i4.2266.
