# Supplementary material for: Trajectory Patterns of Perioperative Anxiety and Depressive Symptoms in Patients With Digestive System Malignancies and Their Impact on Postoperative Outcomes
Source: Actas Esp Psiquiatr. 2026 Aug 15;54(4):1220–33. doi: 10.62641/aep.v54i4.2266 (PMC13571205; doi:10.62641/aep.v54i4.2266)
Supplement: Supplementary file 1 [file ActEsp-54-4-1220-1233-s1.docx]

**Supplementary Material**

*Trajectory Patterns of Perioperative Anxiety and Depressive Symptoms in Patients with Gastrointestinal Malignancies and Their Impact on Postoperative Outcomes*

**Supplementary Table S1. Biomarker Values at T1 and T3 Across Trajectory Groups**

| **Biomarker** | **Time point** | **Resilient (n=120)** | **Recovery (n=109)** | **Chronic (n=56)** | **F** | **P** |
| --- | --- | --- | --- | --- | --- | --- |
| Cortisol (nmol/L) | T1 | 298±54 | 336±62 | 382±70 | 15.84 | <0.001 |
|  | T3 | 326±62 | 398±74 | 468±86 | 24.68 | <0.001 |
| IL-6 (pg/mL) | T1 | 3.6±1.2 | 4.4±1.4 | 5.3±1.8 | 12.46 | <0.001 |
|  | T3 | 4.8±1.6 | 6.4±2.0 | 8.2±2.4 | 28.42 | <0.001 |
| TNF-α (pg/mL) | T1 | 8.4±2.8 | 10.6±3.2 | 12.8±3.8 | 14.26 | <0.001 |
|  | T3 | 10.2±3.4 | 14.2±4.2 | 18.6±5.2 | 22.16 | <0.001 |
| CRP (mg/L) | T1 | 9.6±3.2 | 12.4±4.0 | 15.8±5.4 | 13.82 | <0.001 |
|  | T3 | 28.4±8.6 | 35.2±10.4 | 42.6±12.8 | 18.62 | <0.001 |
| BDNF (ng/mL) | T1 | 14.2±4.0 | 11.8±3.6 | 9.2±3.0 | 11.94 | <0.001 |
|  | T3 | 12.6±3.8 | 9.4±3.2 | 6.8±2.4 | 16.84 | <0.001 |

Abbreviations: T1, 1-3 days preoperatively; T3, postoperative day 7; IL-6, interleukin-6; TNF-α, tumor necrosis factor-α; CRP, C-reactive protein; BDNF, brain-derived neurotrophic factor.

**Supplementary Table S2. Correlations Between HADS Total Scores and Biomarker Levels**

| **Biomarker** | **T1 correlation with HADS total (r)** | **P** | **T3 correlation with HADS total (r)** | **P** |
| --- | --- | --- | --- | --- |
| Cortisol | 0.28 | <0.001 | 0.39 | <0.001 |
| IL-6 | 0.31 | <0.001 | 0.42 | <0.001 |
| TNF-α | 0.27 | <0.001 | 0.36 | <0.001 |
| CRP | 0.24 | 0.001 | 0.33 | <0.001 |
| BDNF | -0.22 | 0.003 | -0.35 | <0.001 |

Note: Correlation analyses were performed between HADS total scores and biomarker levels at T1 and T3. Positive r values indicate that higher HADS total scores were associated with higher biomarker levels, whereas negative r values indicate inverse associations.

**Supplementary Table S3. Distribution of postoperative complication types across trajectory groups**

| **Complication type** | **Resilient (n=120)** | **Recovery (n=109)** | **Chronic (n=56)** | **χ²** | **P** |
| --- | --- | --- | --- | --- | --- |
| Infectious complications, n (%) | 9 (7.5) | 14 (12.8) | 13 (23.2) | 8.55 | 0.014 |
| Anastomotic leakage, n (%) | 2 (1.7) | 4 (3.7) | 5 (8.9) | 5.44 | 0.066 |
| Gastrointestinal dysfunction, n (%) | 4 (3.3) | 8 (7.3) | 9 (16.1) | 9.08 | 0.011 |
| Pulmonary complications, n (%) | 3 (2.5) | 6 (5.5) | 7 (12.5) | 7.21 | 0.027 |
| Postoperative bleeding, n (%) | 1 (0.8) | 2 (1.8) | 3 (5.4) | 3.85 | 0.146 |
| Urinary complications, n (%) | 2 (1.7) | 3 (2.8) | 4 (7.1) | 3.84 | 0.147 |
| Cardiovascular complications, n (%) | 1 (0.8) | 2 (1.8) | 3 (5.4) | 3.85 | 0.146 |

*Note: Complication categories are not mutually exclusive; patients with more than one postoperative complication could be counted in multiple categories.*

**Supplementary Table S4. Distribution of complication severity across trajectory groups**

| **Clavien–Dindo grade*** | **Resilient (n=17)** | **Recovery (n=23)** | **Chronic (n=22)** | **χ²** | **P** |
| --- | --- | --- | --- | --- | --- |
| Overall grade distribution | — | — | — | 14.32 | 0.006 |
| Grade II, n (%) | 15 (88.2) | 19 (82.6) | 10 (45.5) |  |  |
| Grade III, n (%) | 2 (11.8) | 4 (17.4) | 7 (31.8) |  |  |
| Grade IV, n (%) | 0 (0.0) | 0 (0.0) | 5 (22.7) |  |  |

*** *Percentages in Supplementary Table S4 are calculated among patients with at least one postoperative complication within each trajectory group, based on the highest recorded Clavien–Dindo grade per patient.*

**Supplementary Table S5.Distribution of SSRS Scores Across Trajectory Groups**

| **Variable** | **Resilient (n=120)** | **Recovery (n=109)** | **Chronic (n=56)** | **F/χ²** | **P** |
| --- | --- | --- | --- | --- | --- |
| SSRS total score (x̄±s) | 38.6±6.8 | 33.4±7.2 | 27.8±7.6 | 36.48 | <0.001 |
| SSRS total score, median (IQR) | 39 (34–43) | 33 (28–38) | 27 (23–32) | 28.16 | <0.001 |
| SSRS range (min–max) | 22–55 | 18–49 | 14–43 | — | — |
| Low social support, n (%) (SSRS <30) | 16 (13.3) | 34 (31.2) | 28 (50.0) | 27.12 | <0.001 |

**Abbreviations:** SSRS, Social Support Rating Scale; IQR, interquartile range.

**Note:** SRS data were obtained from baseline preoperative assessment. SSRS total scores range from 12 to 66, with higher scores indicating stronger perceived social support. The SSRS <30 threshold was used as a prespecified operational cutoff for identifying relatively reduced social support in this cohort, rather than as a universally validated diagnostic criterion. Group comparisons were performed using one-way ANOVA, Kruskal–Wallis test, or χ² test as appropriate.
